# Serum N-glycomic profiling identifies candidate biomarker panels for assessing coronary artery stenosis severity

**DOI:** 10.1016/j.heliyon.2024.e29443

**Published:** 2024-04-09

**Authors:** Linlin Wu, Haoqi Liu, Xuewen Xu, Chenjun Huang, Yueyue Li, Xiao Xiao, Yueping Zhan, Chunfang Gao

**Affiliations:** a Department of Clinical Laboratory Medicine Center, Yueyang Hospital of Integrated Traditional Chinese and Western Medicine, Shanghai University of Traditional Chinese Medicine, China; b Department of Cardiology, Yueyang Hospital of Integrated Traditional Chinese and Western Medicine, Shanghai University of Traditional Chinese Medicine, China; c Shanghai Cancer Center and Institutes of Biomedical Sciences and Department of Chemistry and NHC Key Laboratory of Glycoconjugates Research, Fudan University, China

**Keywords:** N-glycomic profiling, Biomarkers, Mass spectrometry, Coronary artery stenosis

## Abstract

Stenosis severity may escalate over the course of coronary artery disease (CAD), increasing the risk of death for the patient. Conventionally, the assessment of stenosis degree relies on invasive coronary angiography (ICA), an invasive examination unsuitable for patients in poor physical condition or those with contrast allergies and one that imposes a psychological burden on patients. Although abnormal serum N-glycan profiles have exhibited robust associations with various cardiovascular diseases, including CAD, their potential in diagnosing CAD stenosis remains to be determined. In this study, we performed a comprehensive analysis of serum N-glycome from 132 patients who underwent ICA and 27 healthy controls using MALDI-TOF-mass spectrometry. The patients who underwent ICA examination were categorized into four groups based on stenosis severity: no/mild/moderate/severe stenosis. Twenty-seven N-glycans were directly quantified, and 47 derived glycan traits were obtained. Notably, among these 74 glycan features, 18 exhibited variations across the study groups. Using a combination of least absolute shrinkage and selection operator and logistic regression analyses, we developed five diagnostic models for recognizing stenosis degree. Our results suggested that alterations in serum N-glycosylation modifications might be valuable for identifying stenosis degree and monitoring disease progression in individuals with CAD. It is expected to offer a noninvasive alternative for those who could not undergo ICA because of various reasons. However, the diagnostic potential of serum N-glycan panels as biomarkers requires multicenter, large cohort validation in the future.

## 1 Introduction

Atherosclerosis is a primary driver of coronary artery disease (CAD) and involves the deposition of cholesterol and lipids in the arteries, leading to varying stenosis degrees [1]. With the progression of the disease and the increasing coronary artery stenosis severity, patients may experience chest tightness or chest pain in the anterior region of the heart, indicative of conditions such as CAD and angina pectoris. Without timely intervention, it may affect patients’ cardiac function and potentially pose a threat to their lives. Recently, there has been a significant increase in the incidence of CAD, resulting in a notable increase in mortality and disability [2]. This surge adversely affects the quality of life of patients and their families, imposing a substantial economic burden on society [3]. Currently, the assessment of coronary artery stenosis severity is mainly based on cardiovascular imaging, of which invasive coronary angiography (ICA) is the gold standard [[4], [5], [6]]. However, ICA assessment of coronary artery stenosis degree requires the judgment of at least two experienced physicians, and there is a degree of bias in the results between different doctors [7]. Moreover, ICA is an invasive examination that is both expensive and time-consuming, as well as limited by renal function, making it unsuitable for large-scale screening in the aging population [4]. Hence, the development of a simple and effective method for assessing coronary artery stenosis severity could alleviate patient anxiety, facilitate expanded screening, and enable the timely identification of high-risk patients. This has significant clinical implications for reducing cardiovascular mortality in the community.

There are many traditional risk factors for CAD, including hypertension, hyperlipidemia, hyperglycemia, hyperhomocysteinemia, hyperuricemia, high-salt diet, smoking, alcohol consumption, gender, and genetic factors [1,8]. Moreover, it has been considered to be strongly associated with leukocyte recruitment and arterial inflammation triggered by endothelial cell damage [9]. Several studies have demonstrated that glycans and glycan-binding proteins play an irreplaceable role in the leukocyte recruitment cascade [10,11]. Glycosylation of the adhesion factor GlycA, which responds to inflammatory pathways, has been significantly associated with clinical cardiovascular disease (CVD) [12]. Glycosylation is widespread in serum proteins and influences protein structures, localization, and functions. Mutations in glycosylation genes lead to changes in the expression and activity of serum proteins, including lipoproteins, contributing to a diverse array of clinical manifestations, notably cardiovascular diseases such as atherosclerosis [13,14]. Glycoconjugates are associated with the vasculature, regulate cellular interactions, and can effectively respond to cellular status and the microenvironment [15]. Substantial evidence supports the robust association between abnormal glycosylation and stenosis degree [[16], [17], [18], [19]]. The potential of glycosylation as an early diagnostic and prognostic biomarker for patients with CAD is considerable. Serum is one of the most commonly used liquid biopsy samples in clinical assays. Serum N-glycome contains a wealth of biological information [18,20,21], and its molecular abnormalities can serve as biomarkers for assessing atherosclerotic stenosis degree. This could help clinicians determine the next therapeutic option, thereby reducing the need for ICA examination.

In this study, we used matrix-assisted laser desorption ionization time-of-flight (MALDI-TOF) mass spectrometry (MS) to analyze serum N-glycome. We directly quantified 27 N-glycans, generating 47 derived N-glycan traits. The variations in these 74 features in the study cohorts were assessed. In addition, we combined the least absolute shrinkage and selection operator (LASSO) and logistic regression (LR) modeling to determine the optimal biomarker panel for identifying CAD stenosis degree. During the modeling process, we also integrated additional clinical indicators alongside the glycan features, anticipating the creation of biomarker panels with improved diagnostic results. These biomarkers have the potential to reduce unnecessary ICA testing and to help clinicians determine the necessity for pharmacologic or surgical intervention.

## 2 Materials and methods

### 2.1 Participant selection

Serum samples were collected at Yueyang Hospital of Integrated Traditional Chinese and Western Medicine, Shanghai University of Traditional Chinese Medicine. The inclusion and exclusion criteria for the samples were as follows: All participants were over 45 years old. Patients with CAD were hospitalized and free from diabetes mellitus, malignant tumor history in other organs, rheumatoid arthritis, autoimmune disorders, and allergic reactions to contrast media. The healthy control group was essentially matched to the disease groups in terms of age and gender. They exhibited normal clinical test indicators (blood routine, liver function, renal function, blood lipids, tumor markers, etc.) and had no history of chronic diseases, including tumors, autoimmune diseases, and cardiovascular diseases. A total of 159 samples were collected, comprising 132 patients who underwent ICA examination and 27 healthy individuals. Their age, gender, and relevant clinical characteristics are detailed in Table S1. The patients’ coronary artery stenosis degree was determined by ICA examination and was based on the judgment of the attending physicians. Then, the participant cohort was divided into four subgroups according to coronary artery stenosis severity: 0-CAD (no stenosis), 1-CAD (mild stenosis), 2-CAD (moderate stenosis), and 3-CAD (severe stenosis). All participants signed an informed consent form, and this study was approved by the Review Board and Ethical Committee of Yueyang Hospital (Ethics Approval Number: 2022-125).

### 2.2 Purification of N-glycans from serum samples

N-glycans of serum proteins were released following the procedures described in previously published articles [22,23]. In brief, serum proteins were diluted to a final concentration of 2 μg/μL using 25 mM ammonium bicarbonate (ABC) and subsequently denatured at a constant temperature of 99 °C for 5 min. After cooling naturally to room temperature, 100 units equivalent of PNGase F solution was added and the mixture was incubated at 37 °C overnight. The termination of the reaction was achieved through high-temperature treatment at 99 °C for 10 min. Cotton tips were prepared by filling the base of the tip with 4 ± 0.1 mg of degreased cotton and compacting it. N-glycans were purified from the mixtures using cotton tips, where N-glycans were captured under high-organic-phase conditions and released under high-aqueous-phase conditions [23].

### 2.3 MALDI-TOF MS analysis of serum N-glycans

The lyophilized purified mixture of N-glycans was reconstituted in an ultrapure aqueous solution containing 0.1% trifluoroacetic acid (TFA). The matrix, 2,5-dihydroxybenzoic acid, was dissolved in the mixture solution of 50% ACN/50% H_2_O/0.1% TFA at a final concentration of 10 μg/μL [24]. Sodium chloride solution was added to the aforementioned solution to achieve a final concentration of 1 mmol/L.

MS analysis of serum N-glycans was performed using rapifleX MALDI-TOF MS (Bruker Daltonics, Germany) equipped with a 10 kHz smartbeam 3D laser (Nd:YAG, 355 nm) operating at a frequency of 2000 Hz. Before the samples were tested by MS, external mass calibration was performed using bovine albumin digested peptides, with an error value required to be within 10 ppm. The sample and matrix solution were mixed at a volume ratio of 1:1 (v/v), spotted on an MTP 384 target polished steel plate (Bruker Daltonics, Germany), and allowed to dry naturally. MS spectra were acquired in the positive reflection mode with a laser intensity of 60%. The MS scan range was set at 1000–3000 Da. For each sample, three technical replicates were performed separately, and the data were stored for further analysis.

### 2.4 Data processing and normalization

All raw mass spectra were processed using flexAnalysis software (version 3.4). The list of mass spectra was generated by the same software using manual peak-seeking parameters, which included the mass-to-charge ratio (*m*/*z*), signal-to-noise ratio (S/N), resolution, peak intensity, and peak area. The peak picking parameters were set as follows: centroid algorithm, signal-to-noise threshold = 4, and peak width = 0.2 *m*/*z*. The *m*/*z* values of the peaks were compared with the theoretical *m*/*z* values of the N-glycan compositions using a self-developed Python program, and a manual check was performed to confirm the accurate assignment of the peaks. On the basis of the global distribution of MS quantitative information, we selected 27 stable occurrences of N-glycans for further analysis. The areas of these mass spectral peaks served as the basis for quantitative statistics. To address mass spectral signal bias, we normalized the MS signals of these 27 N-glycans based on the total area of the quantified N-glycans. These N-glycans include monosaccharides such as mannose, galactose, fucose, N-acetylglucosamine (GlcNAc), and sialic acid. Structurally, they encompassed all three N-glycan types: high-mannose, complex, and hybrid types. Moreover, considering the structural characteristics and the charge properties of these 27 glycans, we calculated the percentages of the 47 derived glycan traits. The formulas for the derived glycan traits are detailed in Table S2.

### 2.5 Statistical analysis

Three technical replicates of each serum sample were analyzed to determine the mass spectral peak area of the identified N-glycans. The average of the peak areas from the three replicate assays was calculated and used as the basis for quantification. Differences in serum N-glycan traits among various study cohorts were assessed using the Wilcoxon test (* for p < 0.05; ** for p < 0.01; *** for p < 0.001; **** for p < 0.0001) [25]. The combination of LASSO and LR establishes a diagnostic model to differentiate stenosis degrees [26]. The modeling process was visually represented using software such as GraphPad Prism (version 8.3.0) and R (version 4.3.2). Receiver operating characteristic (ROC) curve analysis was conducted using MedCalc® (version 22.016) to evaluate the discriminatory ability of serum N-glycan features, and the area under the curve (AUC) was calculated to evaluate the classification performance of biomarker panels.

## 3 Results

### 3.1 Clinical characteristics of the participants

**Fig. 1:**
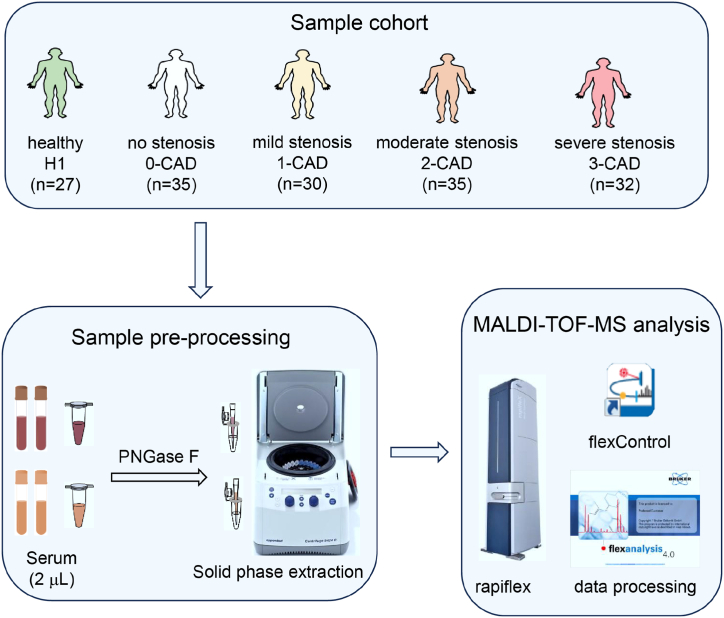
Workflow of serum N-glycan profiling.

The workflow scheme used in this study is illustrated in Fig. 1. The basic characteristics of the subjects enrolled in this study are provided in Table S1. All participants were over 45 years old. Patients (0-CAD, 1-CAD, 2-CAD, and 3-CAD) presenting with symptoms such as shortness of breath and angina pectoris were assessed for clinical indicators of CAD risk factors, including low-density lipoprotein (LDL), triglycerides (TG), high-density lipoprotein (HDL), and total cholesterol (TC), at the hospital. These patients also underwent ICA examination to assess blood vessel conditions, and the ICA results were clinically significant in determining the treatment options. The results of the ICA examination were evaluated by two experienced doctors to determine artery stenosis degree. In this retrospective study, patients were further categorized into four groups based on stenosis degree. The no-stenosis group was categorized as 0-CAD, warranting close monitoring for CAD occurrences; the mild-stenosis group was designated as 1-CAD, necessitating the use of oral statins to manage CAD risk [27]; the moderate-stenosis group was classified as 2-CAD; and the severe-stenosis group was defined as 3-CAD. For the 2-CAD and 3-CAD groups, a combination of oral statins and antiplatelet agents was recommended, with decisions regarding percutaneous coronary intervention based on a comprehensive evaluation of other symptomatic manifestations and lesions [28,29].

### 3.2 Qualitative analysis of serum N-glycans

In this study, N-glycans were released from serum proteins with PNGase F, purified with cotton tips, and analyzed by MALDI-TOF MS. The list of mass spectral peaks was exported using flexAnalysis software, the obtained *m*/*z* was compared with the theoretical *m*/*z* of serum N-glycans using a self-written Python program, and the confirmation of the glycan structures was performed by manual double-checking.

**Fig. 2:**
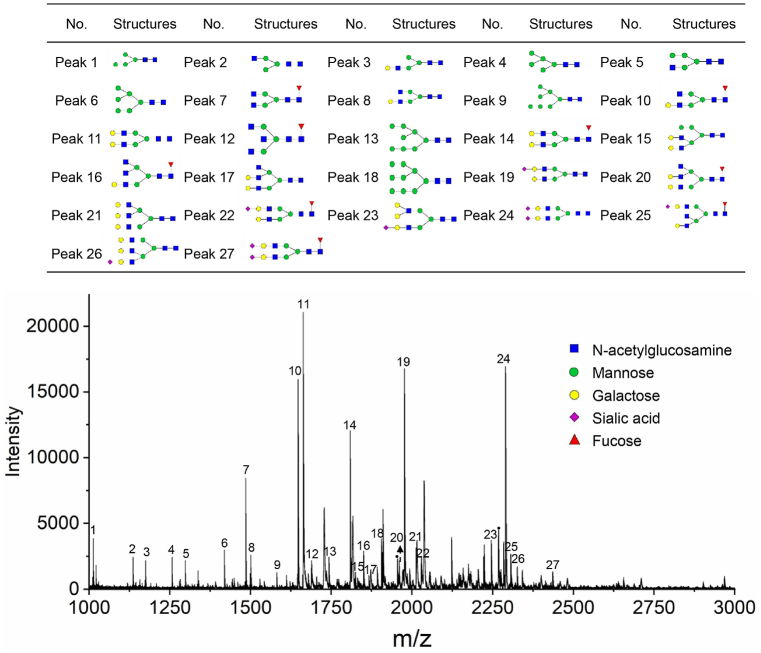
Representative MALDI-TOF MS spectra showing the composition of serum N-glycans.

The glycomic biomarker profiling phase involved 159 samples, each analyzed with three technical replicates, and all 477 (equal to 159 × 3) MS spectra met the quality standards. Following statistical analysis, 27 N-glycans were quantified by the average values of three MS technical replicates, offering simultaneous quantifiable information in over 238 mass spectra (>50%) with coefficients of variation (CV) below 20% [30,31]. Representative MS spectra are depicted in Fig. 2. GlycoWorkbench (version 2.1) software was used to draw the corresponding N-glycan structures [32]. On the basis of these 27 N-glycans, 47 derived N-glycan traits were calculated, and the specific formulas are shown in Table S2.

### 3.3 Alterations in the serum glycan profile

In this study, the CVs of the ratio of neutral glycans to sialic-acid-type glycans were less than 10% in three technical replicate experiments (Fig. S1). Therefore, the error of our experimental method is acceptable for clinical assays. Taking into consideration the nuances of instrument performance and manual operation, the mass spectral signals of N-glycans in this study were normalized by the total area of quantified glycans, which compensated to some extent for the variations in the MS spectral signal intensities among different spectra. Through direct quantification of 27 N-glycans, we calculated the quantitative information of 47 derived glycan traits using the normalized results. Table S2 provides the details of the renumbering from IGP1 to IGP74 and the formulas used to calculate the characteristics of these derived glycan traits.

**Fig. 3:**
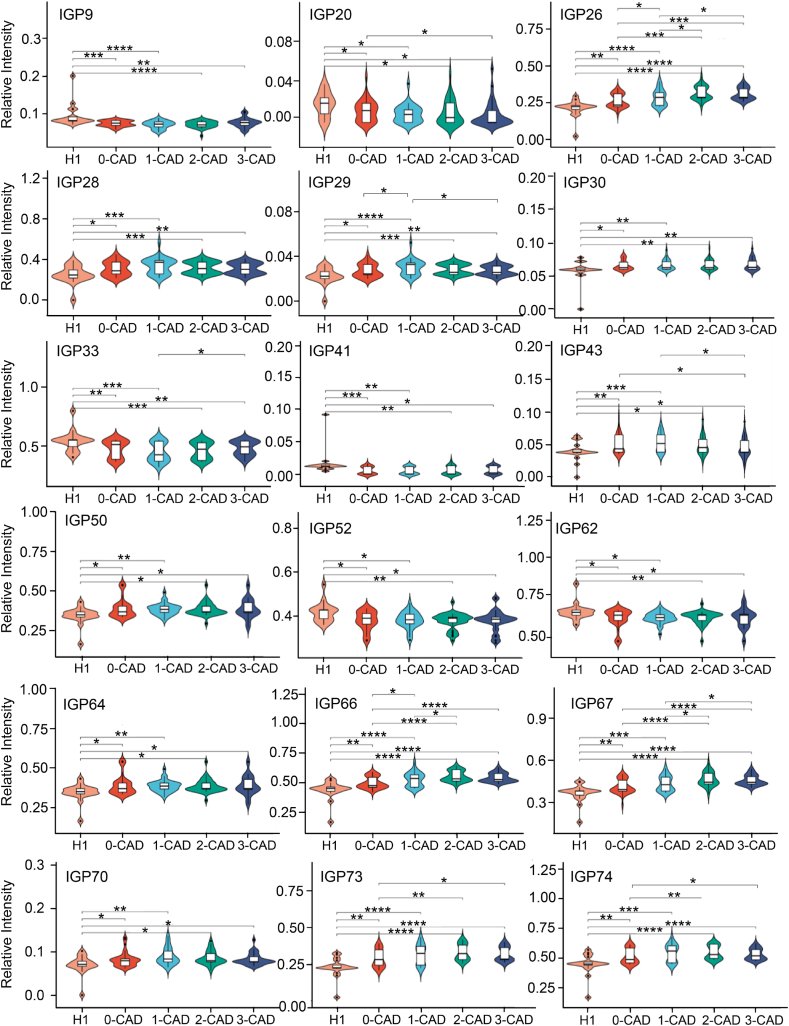
Violin plots of altered serum N-glycan profiles among the H1, 0-CAD (no stenosis), 1-CAD (mild stenosis), 2-CAD (moderate stenosis), and 3-CAD (severe stenosis) groups. * indicates p-value <0.05, ** indicates p-value <0.01, *** indicates p-value <0.001, **** indicates p-value <0.0001.

The results of this study, statistically analyzed using the Wilcoxon test for two-by-two comparisons [33], revealed significant differences in the relative intensities of 3 N-glycans and 15 derived glycan traits among the five study groups (H1, 0-CAD, 1-CAD, 2-CAD, and 3-CAD). The violin box plots in Fig. 3 visualize the pattern of variations for these 18 discrepant IGPs (fold change >1.1 or fold change <0.9 and p < 0.05) [20]. These three serum N-glycans were identified as H5N4, H5N5F1, and H5N4S2. Compared with the H1 group, IGP9 exhibited a significant reduction in the 0-CAD, 1-CAD, 2-CAD, and 3-CAD groups. IGP20 (H5N5F1) exhibited a gradual decrease in abundance corresponding to the progression of stenosis, whereas IGP26 (H5N4S2) exhibited an incremental increase with advancing stenosis. The 15 derived glycan traits were IGP28, IGP29, IGP30, IGP33, IGP41, IGP43, IGP50, IGP52, IGP62, IGP64, IGP66, IGP67, IGP70, IGP73, and IGP74. Detailed N-glycan information is shown in Table S2. In addition, we analyzed the relationship between age and these N-glycans in healthy individuals (over 45 years old) and found no significant correlation (Fig. S2). The results of our analyses indicated that the most significant relative intensity changes observed in the four disease groups and healthy controls were for complex-type N-glycans. This suggested that complex-type N-glycans hold significant promise as biomarkers for distinguishing patients with CAD with varying stenosis degrees. The relationship between complex-type N-glycans and atherosclerosis has been extensively documented in prior studies [34]. Zhou et al. illustrated that hyperphosphatemia in individuals with chronic kidney disease intensified atherosclerosis by promoting the conversion of N-glycans on sterol regulatory element-binding protein and cleavage-activating protein to complex-type N-glycans [35].

### 3.4 Assessment of biomarker candidates for stenosis

To determine panels of potential serum biomarkers, we used LASSO combined with LR analysis to develop the diagnostic model in anticipation of discovering the optimal biomarker panels to discriminate stenosis degree. On the basis of the sample enrolled, the diagnostic model developed in this study can be applied to screening for CAD stenosis in people over 45 years old. Compared with ICA screening, the use of serum biomarkers for screening CAD stenosis offers broader practicality, is cost effective, and alleviates psychological stress for the examinee.

**Fig. 4:**
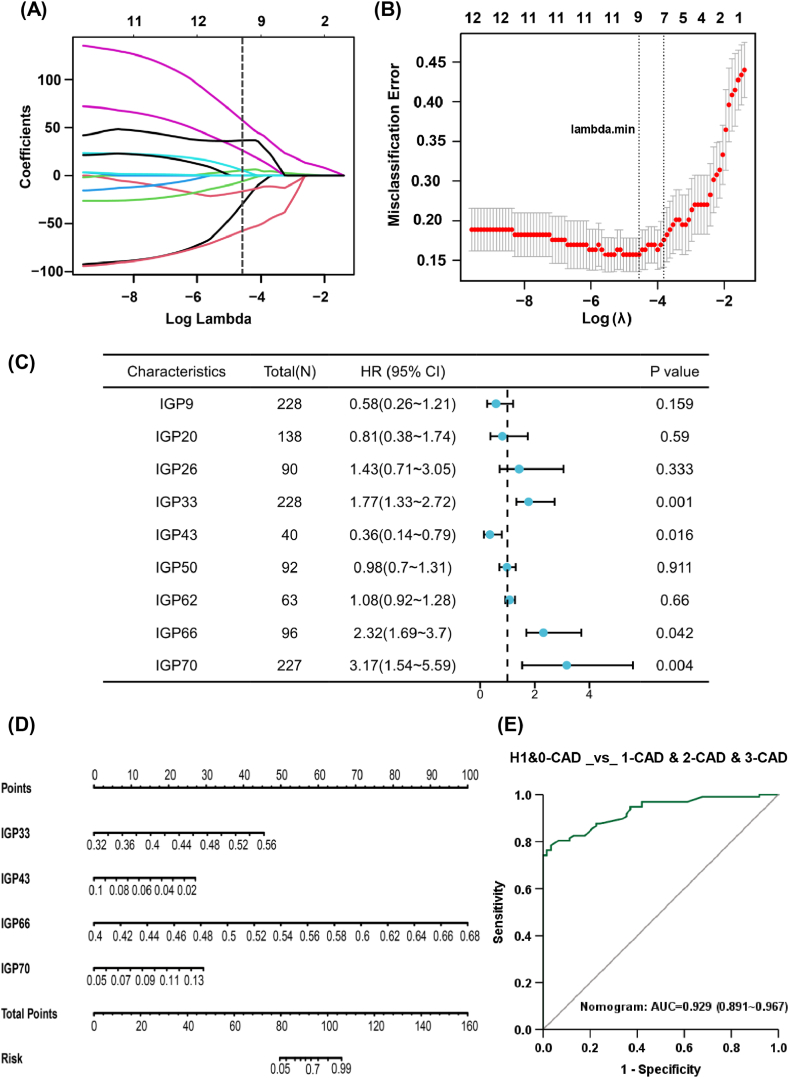
The recognition ability of N-glycome analysis for cases with or without coronary artery stenosis. (A) LASSO coefficient profile plot of the 18 differential glycan features against the log (lambda) sequence. (B) Penalty parameter (lambda) selection by LASSO adopted 10-fold cross-validation. (C) Forest plot of the logistic regression model. (D) Nomogram of the logistic regression model. (E) The receiver operating characteristics (ROC) curves of the nomogram with 95% confidence intervals of the area under the curve (AUC).

We conducted a detailed statistical analysis on the 18 identified differential IGPs (refer to Fig. 3). The goal was to construct an optimal classification model to assess the effectiveness of these differential N-glycan features in distinguishing the arterial stenosis group from the no-stenosis group. In this study, the no-stenosis group included both the H1 and 0-CAD groups in the study cohort. We constructed LASSO regression models using the R package “glmnet” to analyze the diagnostic contributions of these 18 IGPs. LASSO played a crucial role in implementing measures to prevent overfitting, ensuring the robustness and generalizability of the model [36]. As depicted in Fig. 4(A), LASSO employed a penalty function that progressively drove the coefficients of certain factors toward zero, thereby facilitating the development of a more concise model. As illustrated in Fig. 4(B), using 10-fold cross-validation, we selected nine features with nonzero coefficients at Lambda. min for further analysis. Multiple LR analysis was then conducted to develop a diagnostic model for identifying CAD stenosis. Among the nine incorporated features, IGP33, IGP43, IGP66, and IGP70 emerged as specific contributors to the model's performance. The hazard ratios (HRs) of these four features are illustrated in Fig. 4(C): IGP33 (HR: 1.77, 95% CI: 1.33–2.72), IGP43 (HR: 0.36, 95% CI: 0.14–0.79), IGP66 (HR: 2.32, 95% CI: 1.69–3.70), and IGP70 (HR: 3.17, 95% CI: 1.54–5.59). The results of the abovementioned analysis indicated that these four traits served as an optimal diagnostic panel for arterial stenosis, enabling the identification of coronary stenosis in the elderly population. These four traits involved a wide variety of complex-type glycans, including neutral and charged glycans with sialic acid and fucose. The nanogram depicted in Fig. 4(D) results from the integration of these N-glycan features, reflecting the magnitude of the contributions from these four independent risk factors. The ROC curve for the nomogram is shown in Fig. 4(E), with an AUC as high as 0.929 (95% CI: 0.891–0.967), indicating that the discovered biomarker panels have a relatively strong ability to recognize the presence of stenosis.

Subsequently, we used LASSO and LR models to explore the optimal combination of biomarkers for differentiating different CAD stenosis degrees based on the combined characterizations of IGPs and clinical indicators. The LASSO results and the output from the LR are shown in Table S3. This analysis was retrospective, and the study cohort included four patient groups (0-CAD, 1-CAD, 2-CAD, and 3-CAD) that were assessed for stenosis by ICA examination. Considering the reported risk factors, we included clinical indicators associated with CAD stenosis, including age, gender, LDL, HDL, and TC, in LASSO and LR modeling [8,37]. The values of these clinical indicators are presented in Table S1. By applying the modeling approach described above, we developed distinct diagnostic models to recognize varying artery stenosis degrees in patients.

**Fig. 5:**
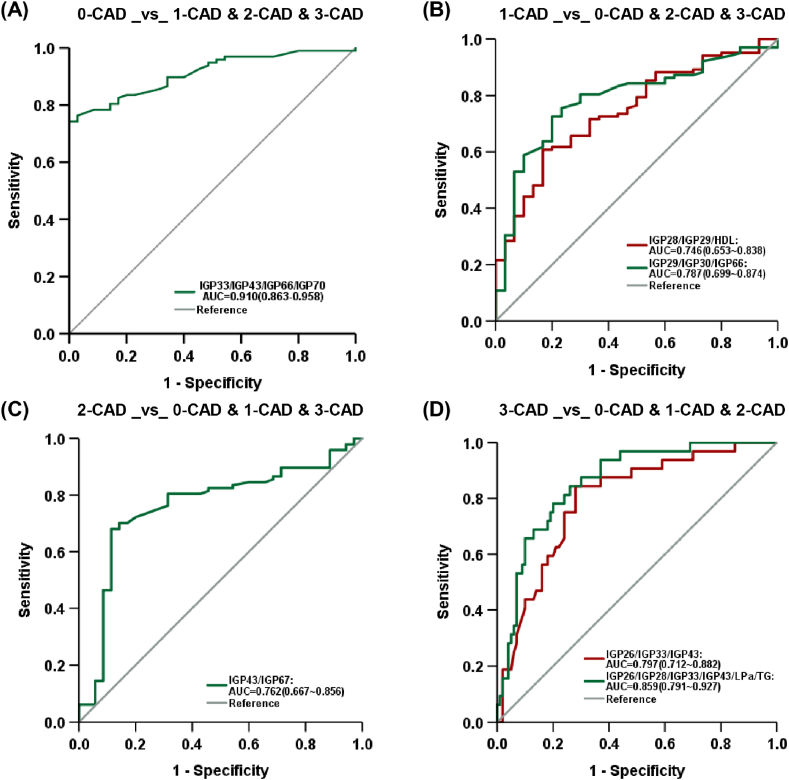
The receiver operating characteristics (ROC) curves of coronary artery stenosis diagnostic models. 0-CAD indicates no stenosis, 1-CAD indicates mild stenosis, 2-CAD indicates moderate stenosis, and 3-CAD indicates severe stenosis.

Through diagnostic modeling, this retrospective study identified optimal biomarker panels and illustrated their diagnostic efficacy using ROC curves (Fig. 5). To enhance the efficiency of identifying subgroups with specific stenosis degrees among the four groups of patients, we compared the modeling results by including only IGPs with those including both IGPs and clinical indicators. We aimed to identify potential biomarker panels for diagnosing CAD stenosis degree. The diagnostic model developed using only IGPs was first evaluated, and the ROC curves showed different AUC areas. The AUC values in Fig. 5(A) (0.910, 95% CI: 0.863–0.958), Fig. 5(B) (0.787, 95% CI: 0.699–0.874), Fig. 5(C) (0.762, 95% CI: 0.667–0.856), and Fig. 5(D) (0.859, 95% CI: 0.712–0.882) represented the model's performance in recognizing varying CAD stenosis degrees (0-CAD, 1-CAD, 2-CAD, and 3-CAD). The above evidence suggested that IGP features were effective in distinguishing subgroups with varying stenosis degrees. When clinical indicators were introduced in addition to the IGPs, two panels of the independent factors ultimately established by the model excluded the clinical indicators. This suggested that IGP features made a more significant contribution than the incorporated clinical indicators in identifying the no-stenosis and moderate-stenosis groups. Although several clinical indicators were reserved during the modeling process for recognizing the mild-stenosis and severe-stenosis groups, they did not have a significant impact on the improvement of specificity and sensitivity. Briefly, the quantitative IGP profile by MS was clinically significant as a diagnostic test for identifying CAD stenosis degree and was anticipated to minimize unnecessary ICA examinations.

## 4 Discussion

The prevalence of CVD in China is continuously increasing, affecting an estimated 330 million individuals. In particular, CAD ranks second, affecting approximately 11.39 million people [38]. CAD is characterized by symptoms of ischemia and hypoxia resulting from the atherosclerosis-induced narrowing of at least 50% of the lumen of coronary arteries that supply blood to the heart [39,40]. Clinical diagnosis of CAD relies on ICA examination. The results of the ICA examination are evaluated by two doctors, who assess that the stenosis of the main coronary artery or the branches of the coronary artery is ≥ 50%, which can be diagnosed as CAD [8]. Although ICA examination is the gold standard for assessing stenosis degree and is one of the most critical references for determining the subsequent steps in patient management, it is essential for diagnosing and planning treatment for patients with CAD. However, it has limitations [5]. ICA examination is a minimally invasive intervention that should be performed by experienced physicians and generally should not be carried out by laboratory technicians. This examination is not suitable for patients with poor kidney function, impaired metabolic function, or allergies to contrast agents [41]. It is noteworthy that ICA examination comes at a relatively high cost and has occasional complications, potentially imposing a physical and mental burden on patients [42,43]. Nevertheless, screening for coronary artery stenosis remains clinically significant despite these drawbacks. Timely diagnosis through ICA can reduce the incidence of adverse events, and early treatment improves patients’ quality of life [44]. Recognizing the need to minimize unnecessary ICA examinations, there is an urgent requirement for developing a simple and rapid noninvasive examination capable of accurately identifying stenosis levels in patients.

Glycosylation modifications have a significant impact on the structures and functions of serum proteins, and aberrant glycosylation plays a key regulatory role in a variety of biological and pathological processes [19,45,46]. This phenomenon makes glycosylation analysis a key tool for gaining insight into disease pathogenesis, discovering potential biomarkers, and driving personalized therapies [9,39,47]. Glycomics as a field of study is dedicated to qualifying or quantifying the glycans in biological samples such as serum and cells. The development of relevant instrumentation and technology has allowed for a more comprehensive and precise understanding of alterations in glycosylation in organisms [20,21,48]. By digging deeper into the quantitative features of glycosylation, researchers can identify disease-specific biomarkers that are essential for developing more precise diagnostic and therapeutic strategies. However, there is a paucity of studies on glycan biomarkers for coronary artery stenosis degree.

In this study, we conducted a comprehensive analysis of serum N-glycan profiles in patients with CAD and healthy individuals using MALDI-TOF MS. The results emphasize that serum N-glycans undergo alterations in patients with CAD as the disease progresses, and these changes vary among different patient subgroups. By employing techniques based on LASSO and LR analysis, we demonstrated that potential biomarker panels of serum N-glycans could effectively differentiate between patients with CAD with varying stenosis degrees and normal controls with high precision. This study directly quantified 27 N-glycans, yielding 47 derived glycan traits, of which 18 glycan features showed differences between the study cohorts. On the basis of these different glycan features, we established diagnostic models and discovered five robust biomarker panels capable of distinguishing H1, 0-CAD, 1-CAD, 2-CAD, and 3-CAD. This study explored the correlation between varying stenosis degrees and glycan features, with results suggesting that serum N-glycans could serve as potential biomarkers for assessing CAD stenosis degree. Further validation of its diagnostic efficacy is required in a multicenter clinical cohort. Once these biomarkers are validated thoroughly, we can assay the N-glycan features in human serum and can apply these models to assess CAD stenosis severity. For example, physicians can assess whether a patient's CAD stenosis has reached the 2-CAD level by combining quantitative information on IGP43 and IGP67 with the model in Fig. 5(C). After treatment, the method can also be used to assess whether stenosis severity has improved. Serum diagnostic markers make it unnecessary for patients to undergo multiple ICA examinations.

Serum, being one of the most commonly used sources of liquid biomarkers, coupled with the rapid profiling of N-glycans using MALDI-TOF MS, is expected to offer a noninvasive alternative for those who cannot undergo ICA because of various reasons. Serum N-glycan panels as biomarkers have greater generalizability and can be promoted to expand screening for CAD stenosis degree in high-risk populations such as the elderly. Furthermore, this study may enhance our understanding of the role of serum glycosylation, thereby opening new avenues for grading stenosis degree or even predicting treatment prognosis.

## Data availability

Data included in the article/supp. Material/referenced in the article.

## Ethics statement

Written informed consent was obtained from the individual(s) for the publication of any potentially identifiable images or data included in this article. Ethical approval for this study was obtained from the Review Board and Ethical Committee of Yueyang Hospital (Ethics Approval Number: 2022-125; Ethics Approval Date: 2022.12.22).

## Funding statement

This research was supported by the 10.13039/501100001809National Science Foundation of China under grant number 82372321, 10.13039/501100014137Shanghai Shenkang Hospital Development Center Project under grant number SHDC2023CRT015, and Science and Technology Development Fund of 10.13039/501100010876Shanghai University of Traditional Chinese Medicine under grant number 23KFL113.

## CRediT authorship contribution statement

**Linlin Wu:** Writing – review & editing, Writing – original draft, Visualization, Data curation. **Haoqi Liu:** Resources. **Xuewen Xu:** Resources. **Chenjun Huang:** Methodology. **Yueyue Li:** Resources. **Xiao Xiao:** Resources. **Yueping Zhan:** Resources. **Chunfang Gao:** Writing – review & editing.

## Declaration of competing interest

The authors declare that they have no known competing financial interests or personal relationships that could have appeared to influence the work reported in this paper.

## References

[1] Freeman A.M., Raman S.V., Aggarwal M., Maron D.J., Bhatt D.L., Parwani P., Osborne J., Earls J.P., Min J.K., Bax J.J., Shapiro M.D. (2023). Integrating coronary atherosclerosis burden and progression with coronary artery disease risk factors to guide therapeutic decision making[J]. Am. J. Med..

[2] Gaudio L.T., Veltri P., De Rosa S., Indolfi C., Fragomeni G. (2020). Model and application to support the coronary artery diseases (CAD): development and testing[J]. Interdiscipl. Sci. Comput. Life Sci..

[3] Zhou M., Wang H., Zeng X., Yin P., Zhu J., Chen W., Li X., Wang L., Wang L., Liu Y., Liu J., Zhang M., Qi J., Yu S., Afshin A., Gakidou E., Glenn S., Krish V.S., Miller-Petrie M.K., Mountjoy-Venning W.C., Mullany E.C., Redford S.B., Liu H., Naghavi M., Hay S.I., Wang L., Murray C.J.L., Liang X. (2019). Mortality, morbidity, and risk factors in China and its provinces, 1990-2017: a systematic analysis for the Global Burden of Disease Study 2017[J]. Lancet.

[4] Abensur Vuillaume L., Gentilhomme C., Weber S., Ouamara N., Bayard J., Valla M., Khalife K., Goetz C., Guler N. (2022). Effectiveness of hypnosis for the prevention of anxiety during coronary angiography (HYPCOR study): a prospective randomized study[J]. BMC Compl. Alternative Med..

[5] Chorianopoulos E. (2018). Current value of diagnostic coronary angiography[J]. Zeitschrift fur Herz Thorax und Gefasschirurgie.

[6] Gupta H., Spanopoulous B., Lubat E., Krinsky G., Rutledge J., Fortier J.H., Grau J., Tayal R. (2023). Real-world approach to comprehensive artificial intelligence-aided CT evaluation of coronary artery disease in 530 patients: a retrospective study[J]. Heliyon.

[7] Messner M., Meyers S.E., Kemp W.L. (2020). A comparison of coronary artery stenosis estimates made by forensic pathologists and medical students[J]. J. Forensic Sci..

[8] Perpetuo L., Barros A.S., Dalsuco J., Nogueira-Ferreira R., Resende-Goncalves P., Falcao-Pires I., Ferreira R., Leite-Moreira A., Trindade F., Vitorino R. (2022). Coronary artery disease and aortic valve stenosis: a urine proteomics study[J]. Int. J. Mol. Sci..

[9] Pu Q., Glycosyltransferases C. Yu (2014). Glycosylation and atherosclerosis[J]. Glycoconj. J..

[10] F. Krautter, A. J. Iqbal. Glycans and glycan-binding proteins as regulators and potential targets in leukocyte recruitment[J]. Front. Cell Dev. Biol.,92021) 624082.. 10.3389/fcell.2021.624082 PMC7890243 33614653

[11] C. Mauersberger, J. Hinterdobler, H. Schunkert, T. Kessler, H. B. Sager. Where the actionis-leukocyte recruitment in atherosclerosis[J]. Frontiers in Cardiovascular Medicine,82021) 813984.. 10.3389/fcvm.2021.813984 PMC8787128 35087886

[12] Benson E.A., Tibuakuu M., Zhao D., Akinkuolie A.O., Otvos J.D., Duprez D.A., Jacobs D.R., Mora S., Michos E.D. (2018). Associations of ideal cardiovascular health with GlycA, a novel inflammatory marker: the Multi-Ethnic Study of Atherosclerosis[J]. Clin. Cardiol..

[13] Eckardt V., Weber C., von Hundelshausen P. (2019). Glycans and glycan-binding proteins in atherosclerosis[J]. Thromb. Haemostasis.

[14] Pirillo A., Svecla M., Catapano A.L., Holleboom A.G., Norata G.D. (2021). Impact of protein glycosylation on lipoprotein metabolism and atherosclerosis[J]. Cardiovasc. Res..

[15] J. Zhang, N. Ju, X. Yang, L. Chen, C. Yu. The alpha1,3-fucosyltransferase FUT7 regulates IL-1 beta-induced monocyte-endothelial adhesion via fucosylation of endomucin[J].Life Sci. 1922018) 231-237.. 10.1016/j.lfs.2017.11.017 29138114

[16] Menni C., Gudelj I., Macdonald-Dunlop E., Mangino M., Zierer J., Besic E., Joshi P.K., Trbojevic-Akmacic I., Chowienczyk P.J., Spector T.D., Wilson J.F., Lauc G., Valdes A.M. (2018). Glycosylation profile of immunoglobulin G Is cross-sectionally associated with cardiovascular disease risk score and subclinical atherosclerosis in two independent cohorts[J]. Circ. Res..

[17] D. Kaur, H. Khan, A. K. Grewal, T. G. Singh. Glycosylation: a new signaling paradigm for the neurovascular diseases[J]. Life Sci.,3362023) 122303.. 10.1016/j.lfs.2023.122303 38016576

[18] J. Li, Y. Qiu, C. Zhang, H. Wang, R. Bi, Y. Wei, Y. Li, B. Hu. The role of protein glycosylation in the occurrence and outcome of acute ischemic stroke[J].Pharmacol. Res., 1912023) 106726.. 10.1016/j.phrs.2023.106726 36907285

[19] Suzuki H., Chikada M., Yokoyama M.K., Kurokawa M.S., Ando T., Furukawa H. (2016). Aberrant glycosylation of lumican in aortic valve stenosis revealed by proteomic analysis.[J]. Int. Heart J..

[20] Wang Y., Liu S., Li J., Yin T., Liu Y., Wang Q., Liu X., Cheng L. (2023). Comprehensive serum N-glycan profiling identifies a biomarker panel for early diagnosis of non-small-cell lung cancer[J]. Proteomics.

[21] Huang C., Xu X., Wang M., Xiao X., Cheng C., Ji J., Fang M., Gao C. (2021). Serum N-glycan fingerprint helps to discriminate intrahepatic cholangiocarcinoma from hepatocellular carcinoma[J]. Electrophoresis.

[22] Wei L., Cai Y., Yang L., Zhang Y., Lu H. (2018). Duplex stable isotope labeling (DuSIL) for simultaneous quantitation and distinction of sialylated and neutral N-glycans by MALDI-MS[J]. Anal. Chem..

[23] Y. Peng, J. Lv, L. Yang, D. Wang, Y. Zhang, H. Lu. A streamlined strategy for rapid and selective analysis of serum N-glycome[J].Anal. Chim. Acta, 10502019) 80-87.. 10.1016/j.aca.2018.11.002 30661594

[24] Peng Y., Gu B., Sun Z., Li Y., Zhang Y., Lu H. (2021). Linkage-selective derivatization for glycosylation site- and glycoform-specific characterization of sialic acid isomers using mass spectrometry[J]. Chem. Commun..

[25] Kvist M., Välimaa L., Harel A., Malmi S., Tuomisto A. (2023). Glycans as potential diagnostic markers of traumatic brain injury in children[J]. Diagnostics.

[26] Kim H., Kim H. (2018). Functional logistic regression with fused lasso penalty[J]. J. Stat. Comput. Simulat..

[27] Balk E.M., Karas R.H., Jordan H.S., Kupelnick B., Chew P., Lau J. (2004). Effects of statins on vascular structure and function: a systematic review[J]. Am. J. Med..

[28] Redberg R.F. (2018). Overuse of percutaneous coronary interventions[J]. JAMA Intern. Med..

[29] Bhatt D.L. (2018). Percutaneous coronary intervention in 2018[J]. JAMA.

[30] Lee S.B., Bose S., Ahn S.H., Son B.H., Ko B.S., Kim H.J., Chung I.Y., Kim J., Lee W., Ko M.S., Lee K., Chang S., Park H.S., Lee J.W., Kim D.C. (2020). Breast cancer diagnosis by analysis of serum N-glycans using MALDI-TOF mass spectroscopy[J]. PLoS One.

[31] P. M. Angel, R. R. Drake, Y. Park, C. L. Clift, C. West, S. Berkhiser, G. Hardiman, A. S. Mehta, D. P. Bichell, Y. R. Su. Spatial N-glycomics of the human aortic valve in development and pediatric endstage congenital aortic valve stenosis[J].J. Mol. Cell. Cardiol., 1542021) 6-20.. 10.1016/j.yjmcc.2021.01.001 PMC8722354 33516683

[32] Ceroni A., Maass K., Geyer H., Geyer R., Dell A. (2008). GlycoWorkbench: a tool for the computer assisted annotation of mass spectra of glycans[J]. J. Proteome Res..

[33] M. Kvist, L. Välimaa, A. Harel, J. P. Posti, M. Rahi, I. Saarenpää, M. Visuri, A. Östberg, J. Rinne. Glycans as potential diagnostic markers of traumatic brain injury[J].Brain Sci., 112021) 1480.. 10.3390/brainsci11111480 PMC8615782 34827479

[34] Qian Y., Zhang X., Zhou L., Yun X., Xie J., Xu J., Ruan Y., Ren S. (2012). Site-specific N-glycosylation identification of recombinant human lectin-like oxidized low density lipoprotein receptor-1 (LOX-1)[J]. Glycoconj. J..

[35] Ryu H., Lim H., Choi G., Park Y.-J., Cho M., Na H., Ahn C.W., Kim Y.C., Kim W.-U., Lee S.-H., Chung Y. (2018). Atherogenic dyslipidemia promotes autoimmune follicular helper T cell responses via IL-27[J]. Nat. Immunol..

[36] Han D., He X. (2023). Screening for biomarkers in age-related macular degeneration[J]. Heliyon.

[37] Thai P.V., Tien H.A., Van Minh H., Valensi P. (2020). Triglyceride glucose index for the detection of asymptomatic coronary artery stenosis in patients with type 2 diabetes[J]. Cardiovasc. Diabetol..

[38] Hu S.S., Wang Z.W. (2023). Overview of report on cardiovascular health and diseases in China 2022[J]. Chinese Journal of Cardiovascular Research.

[39] Krishnan S., Huang J., Lee H., Guerrero A., Berglund L., Anuurad E., Lebrilla C.B., Zivkovic A.M. (2015). Combined high-density lipoprotein proteomic and glycomic profiles in patients at risk for coronary artery disease[J]. J. Proteome Res..

[40] Satoh K., Fukumoto Y., Sugimura K., Miura Y., Aoki T., Nochioka K., Tatebe S., Miyamichi-Yamamoto S., Shimizu T., Osaki S., Takagi Y., Tsuburaya R., Ito Y., Matsumoto Y., Nakayama M., Takeda M., Takahashi J., Ito K., Yasuda S., Shimokawa H. (2013). Plasma cyclophilin a is a novel biomarker for coronary artery disease[J]. Circ. J..

[41] Ford T.J., McEntegart M., Berry C., Oldroyd K.G. (2018). Arterial access for invasive coronary angiography: the ‘left backhander’[J]. Heart Lung Circ..

[42] Bingen B.O., Al Amri I., Oliveri F., Cabezas J.M. (2023). Invasive angiography in elucidating ambiguous shunt anatomy[J]. JACC Cardiovasc. Interv..

[43] G. Silva, C. E. Guerreiro, P. G. Teixeira, P. R. Queiros, M. R. Silva. Feasibility of coronary angiography after TAVR[J]. Eur. Heart J. 422021) 2082..

[44] Knol W.G., Wahadat A.R., Roos-Hesselink J.W., Van Mieghem N.M., Tanis W., Bogers A.J.J.C., Budde R.P.J. (2021). Screening for coronary artery disease in early surgical treatment of acute aortic valve infective endocarditis[J]. Interact. Cardiovasc. Thorac. Surg..

[45] Li L., Qu C., Wu X., Dai J., Lu Y., Gong Y., You R., Liu Y. (2018). Patterns and levels of platelet glycosylation in patients with coronary heart disease and type 2 diabetes mellitus[J]. J. Thromb. Thrombolysis.

[46] L. Yu, J. Peng, C. Mineo. Lipoprotein sialylation in atherosclerosis: lessons from mice[J]. Front. Endocrinol.,132022) 953165.. 10.3389/fendo.2022.953165 PMC9498574 36157440

[47] Xu Y.X., Ashline D., Liu L., Tassa C., Shaw S.Y., Ravid K., Layne M.D., Reinhold V., Robbins P.W. (2015). The glycosylation-dependent interaction of perlecan core protein with LDL: implications for atherosclerosis[J]. JLR (J. Lipid Res.).

[48] X. FengBaiMaYangJin, X. Mo, F. Zhang, W. Hu, Z. Feng, T. Zhang, L. Wei, H. Lu. IgG glycomic profiling identifies potential biomarkers for diagnosis of echinococcosis[J]. J. Chromatogr., B: Anal. Technol. Biomed. Life Sci.,12272023) 123838.. 10.1016/j.jchromb.2023.123838 37540936

