# Supplementary material for: Serum N-glycomic profiling identifies candidate biomarker panels for assessing coronary artery stenosis severity
Source: Heliyon. 2024 Apr 9;10(7):e29443. doi: 10.1016/j.heliyon.2024.e29443 (PMC11021961; doi:10.1016/j.heliyon.2024.e29443)
Supplement: Multimedia component 1 [file mmc1.docx]

**Figure S1.** Ratio of the relative content of neutral glycans to sialic-acid-type glycans and coefficients of variation of three technical replicates.

**Figure S2.** Violin plots of serum N-glycan profiles among the healthy individuals.
